# Identification of *sdiA*-regulated genes in a mouse commensal strain of *Enterobacter cloacae*

**DOI:** 10.3389/fcimb.2015.00047

**Published:** 2015-05-27

**Authors:** Anice Sabag-Daigle, Jessica L. Dyszel, Juan F. Gonzalez, Mohamed M. Ali, Brian M. M. Ahmer

**Affiliations:** ^1^Department of Microbial Infection and Immunity, The Ohio State UniversityColumbus, OH, USA; ^2^Center for Microbial Interface Biology, The Ohio State UniversityColumbus, OH, USA; ^3^Department of Microbiology, The Ohio State UniversityColumbus, OH, USA; ^4^Department of Medical Microbiology and Immunology, Faculty of Medicine, Mansoura UniversityMansoura, Egypt

**Keywords:** SdiA, LuxR solo, *Enterobacter*, regulon, acylhomoserine lactone, transposon mutagenesis, suicide vector, quorum sensing

## Abstract

Many bacteria determine their population density using quorum sensing. The most intensively studied mechanism of quorum sensing utilizes proteins of the LuxI family to synthesize a signaling molecule of the acylhomoserine lactone (AHL) type, and a protein of the LuxR family to bind AHL and regulate transcription. Genes regulated by quorum sensing often encode functions that are most effective when a group of bacteria are working cooperatively (e.g., luminescence, biofilm formation, host interactions). Bacteria in the *Escherichia, Salmonella, Klebsiella*, and *Enterobacter* genera do not encode an AHL synthase but they do encode an AHL receptor of the LuxR family, SdiA. Instead of detecting their own AHL synthesis, these organisms use SdiA to detect the AHLs synthesized by other bacterial species. In this study, we used a genetic screen to identify AHL-responsive genes in a commensal *Enterobacter cloacae* strain that was isolated from a laboratory mouse. The genes include a putative type VI secretion system, *copA* (a copper transporter), and *fepE* (extends O-antigen chain length). A new transposon mutagenesis strategy and suicide vectors were used to construct an *sdiA* mutant of *E. cloacae*. The AHL-responsiveness of all fusions was entirely *sdiA*-dependent, although some genes were regulated by *sdiA* in the absence of AHL.

## Introduction

Many bacteria monitor their population density (often called quorum sensing) as one of many inputs to gene regulation. The genes regulated by quorum sensing are often those that provide maximal benefit when expressed simultaneously throughout a population (Schuster et al., 2013). The classic example is the expression of luciferase by *Vibrio fischeri*, in which luminescence is most effective when the entire population participates (Hastings and Greenberg, 1999). Other examples include the expression of conjugation functions, biofilm formation, or various aspects of host interaction (Rutherford and Bassler, 2012).

The most intensively studied type of quorum sensing utilizes a protein of the LuxI family to synthesize a signaling molecule of the acylhomoserine lactone (AHL) type (Schaefer et al., 1996). Accumulation of the AHL results in its detection by a transcription factor of the LuxR family. In the case of *V. fischeri*, the LuxR-AHL complex binds upstream of the *luxICDABEG* operon to activate the expression of luciferase (Choi and Greenberg, 1991; Hanzelka and Greenberg, 1995). Thus, the population of bacteria cooperate to create light and illuminate their host, the squid *Euprymna scolopes* (Chun et al., 2008; Miyashiro and Ruby, 2012).

Homologous LuxI/LuxR regulatory systems have been identified in numerous Proteobacteria (Case et al., 2008). Some bacteria that live in mammalian intestinal tracts encode AHL synthases, although AHLs themselves have not yet been demonstrated to be present in this environment (Swearingen et al., 2012). Interestingly, a LuxR homolog, SdiA, has been identified in the *Enterobacteriaceae*, including the genera *Escherichia, Salmonella, Enterobacter, Klebsiella*, and *Citrobacter*. However, these organisms do not encode a cognate AHL synthase (Smith and Ahmer, 2003; Sabag-Daigle and Ahmer, 2012). Instead, it has been shown that SdiA of *E. coli* and *Salmonella enterica* detect the AHLs produced by other species of bacteria (Michael et al., 2001; Smith and Ahmer, 2003; Dyszel et al., 2010a,b; Sperandio, 2010a; Soares and Ahmer, 2011; Sheng et al., 2013). In *Salmonella enterica* serovar Typhimurium, SdiA positively regulates two loci, (1) the *rck* (resistance to complement killing) operon located on the virulence plasmid, pSLT (Ahmer et al., 1998; Michael et al., 2001; Smith and Ahmer, 2003; Abed et al., 2014); and (2) *srgE* (*sdiA*-regulated gene), a single gene horizontal acquisition that encodes an effector protein that is secreted by type III secretion system 2 (T3SS2) (Smith and Ahmer, 2003; Habyarimana et al., 2014). SdiA in EHEC functions to activate expression of genes involved in the glutamate-dependent acid resistance system (*gad)* and has also been found to repress the expression of flagella genes and the enterocyte effacement (LEE) locus (Van Houdt et al., 2006; Lee et al., 2008; Nikaido et al., 2008; Dyszel et al., 2010b; Hughes et al., 2010; Nguyen and Sperandio, 2012; Nguyen et al., 2013; Sheng et al., 2013). Competition assays in cattle of wild-type EHEC and an isogenic *sdiA* mutant indicate a defect of the *sdiA* mutant in colonization of rumen and the recto-anal junction (RAJ) (Hughes et al., 2010; Sheng et al., 2013). This phenotype was shown to correlate with lack of *gad* activation in the rumen and a failure to repress the LEE locus in the RAJ in the absence of *sdiA* (Hughes et al., 2010; Nguyen et al., 2013). In a plant-associated isolate of *Enterobacter*, an *sdiA* mutation derepresses the *csgBAC* operon leading to an overproduction of curli fimbrae (Shankar et al., 2012). The *sdiA* mutant has increased root colonization and biofilm formation correlating with the increased expression of curli adhesion molecules (Shankar et al., 2012).

We wanted to study the role of *sdiA* in a commensal member of the murine microbiota. Laboratory strains of *E. coli* K-12 and EHEC do not colonize mice well. Commensal strains of *E. coli* recovered from mice are very rare in the literature, and during microbiome studies *E. coli* has been found to be rare or non-existent in mice depending on strain and vendor. In this study, we performed a genetic screen to identify AHL-responsive genes of an *Enterobacter cloacae* strain that was isolated from laboratory mice (Ali et al., 2014). We utilized a transposon to create chromosomal *luxCDABE* fusions in a wild-type background, with *sdiA* at its natural position in the chromosome. We screened these fusions to identify those that are AHL-responsive. A new suicide vector and novel mutagenesis strategy were then used to mutate *sdiA* in each fusion strain. The AHL-responsiveness of all of the fusions was entirely *sdiA*-dependent, but a few genes were regulated by *sdiA*, including one gene repressed by *sdiA*, largely in the absence of AHLs. This ligand-independent activity of SdiA has important implications for our understanding of the role of this LuxR solo (Patankar and Gonzalez, 2009; Subramoni and Venturi, 2009).

## Materials and methods

### Bacterial strains and media

Bacterial strains are listed in Table 1. Bacteria were routinely grown in Luria-Bertani (LB) broth or on LB agar unless otherwise stated. LB motility agar was also used (LB broth + 0.3% agar). Chloramphenicol (cam), kanamycin (kan), tetracycline (tet), ampicillin (amp), and nalidixic acid (nal) were used at 20, 50, 10, 200, and 50 μg/ml, respectively. *N*-(3-oxo-hexanoyl)-L-homoserine lactone (oxoC6) was obtained from Sigma-Aldrich and dissolved in ethyl acetate that had been acidified by the addition of 0.1 ml glacial acetic acid per liter (EA) (Pearson et al., 1994). The stock concentration of oxoC6 was 1 mM and it was used at a final concentration of 1 μM. Solvent controls were performed by using EA alone at 0.1%.

**Table 1:** **Strains and plasmids**.

**Strain or plasmid**	**Genotype**	**Source or references**
**STRAINS**		
14028	Wild-type *Salmonella enterica* subspecies *enterica* serovar Typhimurium	American type culture collection
AL4001	*E. coli* BA4000 *gadW*4001::mTn5*luxkan2*	Dyszel et al., 2010b
BA4000	Nal^R^ resistant mutant of *E. coli* BW25113	Dyszel et al., 2010b
BW20767	*E. coli leu*-63::IS10 *recA*1 *creC*510 *hsdR*17 *endA*1 *zbf*-5 *uidA*(Δ *Mlu*I)::*pir*+ *thi* RP4-2-*tet*::Mu-1*kan*::Tn7	Metcalf et al., 1996
JLD400	*Enterobacter cloacae* mouse isolate	Ali et al., 2014
JLD401	*Enterobacter cloacae* mouse isolate, Nal^R^	This study
JLD500	JLD401 ENC_40870::mTn5*luxkan2*	This study
JLD501	JLD401 ENC_10940::mTn5*luxkan2*	This study
JLD502	JLD401 ENC_30820::mTn5*luxkan2*	This study
JLD504	JLD401 ENC_11220IG::mTn5*luxkan2*	This study
JLD505	JLD401 ENC_07270::mTn5*luxkan2*	This study
JLD506	JLD401 ENC_02820::mTn5*luxkan2*	This study
JLD507	JLD401 ENC_22440::mTn5*luxkan2*	This study
JLD508	JLD401 ENC_22440::mTn5*luxkan2*. ENC_22440 is a *copA* homolog	This study
JLD509	JLD401 ENC_30820::mTn5*luxkan2*	This study
JLD511	JLD401 ENC_10940::mTn5*luxkan2*	This study
JLD513	JLD401 ENC_40870::mTn5*luxkan2*	This study
JLD514	JLD401 ENC_40870::mTn5*luxkan2*	This study
JLD515	JLD401 ENC_14970IG::mTn5*luxkan2*	This study
JLD516	JLD401 ENC_14970IG::mTn5*luxkan2*	This study
JLD517	JLD401 ENC_40870::mTn5*luxkan2*	This study
JLD518	JLD401 ENC_40870::mTn5*luxkan2*	This study
JLD519	JLD401 ENC_40870::mTn5*luxkan2*	This study
JLD800	AL4001 *sdiA*271::*cam*	Dyszel et al., 2010b
ASD401	JLD401 *sdiA32*::mTn5-FC	This study
ASD500	JLD401 ENC_40870::mTn5*luxkan2 sdiA32*::mTn5-FC	This study
ASD501	JLD401 ENC_10940::mTn5*luxkan2 sdiA32*::mTn5-FC	This study
ASD502	JLD401 ENC_30820::mTn5*luxkan2 sdiA32*::mTn5-FC	This study
ASD504	JLD401 ENC_11220IG::mTn5*luxkan2 sdiA32*::mTn5-FC	This study
ASD505	JLD401 ENC_07270::mTn5*luxkan2 sdiA32*::mTn5-FC	This study
ASD506	JLD401 ENC_02820::mTn5*luxkan2 sdiA32*::mTn5-FC	This study
ASD508	JLD401 ENC_22440::mTn5*luxkan2 sdiA32*::mTn5-FC	This study
ASD515	JLD401 ENC_14970IG::mTn5*luxkan2 sdiA32*::mTn5-FC	This study
ASD708	BW20767 + pASD708	This study
**PLASMIDS**		
pUT mTn5lux kan2	Suicide vector, ori R6K, mini-Tn5 Km2 *luxCDABE* transposon, mob+ (RP4) Amp^R^ Kan^R^	Winson et al., 1998
pMO197	Suicide vector, *oriT oriV sacB* TcR, *ccdB*, Tet^R^	This study
pMO704	Suicide vector, *oriT oriV sacB* TcR, *ccdB*, Amp^R^	This study
pASD704	pCR8/GW/TOPO, *E. cloacae sdiA*	This study
pASD706	pCR8/GW/TOPO, *E. cloacae sdiA32*::mTn5-FC	This study
pASD708	pMO197, *E. cloacae sdiA32*::mTn5-FC	This study
pCR8/TOPO/GW	Cloning vector, Spec^R^	Invitrogen
pJNS25	P*_srgE_*-*luxCDABE* p15A ori Tet^R^	Smith and Ahmer, 2003

### Constructing transposon based luciferase fusions and screening for AHL responsiveness in *E. cloacae*

Transposon mutagenesis was performed by mating BW20767+pUTmTn*5luxkan2* (Winson et al., 1998) and JLD401, a spontaneous nalidixic acid resistant mutant of *Enterobacter cloacae* strain JLD400. The two strains were plated on LB plates at 37°C overnight. Cells were then scraped with sterile PBS and plated on LB kan nal. 10,000 single colonies were patched into 96-well plates with 0.3% motility agar in the presence of oxoC6 or the solvent control, EA, at 37°C for 9 h. Plates were read with a Wallac Victor3 (Perkin Elmer) plate reader. Those wells that had greater than 3-fold difference after 9 h were streaked for isolation on LB kan nal plates at 37°C overnight. For confirmation, one colony from each plate was inoculated into LB kan nal broth or 0.3% motility agar in 96-well format in the presence of oxoC6, or the solvent control, EA. Plates were read on the Victor plate reader every 3 h. Those fusions that demonstrated greater than 2.5-fold AHL-dependent induction after 9 h were saved for future studies.

### Identification of transposon insertion sites

Genomic DNA was isolated from overnight cultures of the transposon insertion mutants using the GenElute™ Bacterial Genomic DNA Isolation kit (Sigma Aldrich, St. Louis, MO). The transposon insertion site in the genomic DNA was sequenced using Sanger sequencing with two different primers, BA247 and BA1090 (Table 2). Both sequencing primers bind within the *luxC* coding region oriented out of the transposon. Oligonucleotides were synthesized by Integrated DNA Technologies (IDT, Coralville, IA). DNA sequencing was performed by the Plant Microbe Genomics Facility at The Ohio State University. The sequence adjacent to the transposon insertion site was used for BLASTN searches using the BLAST program at the National Center for Biotechnology Information (NCBI).

**Table 2:** **Oligonucleotides**.

**Name**	**Sequence**	**Description**
BA247	GAGTCATTCAATATTGGCAGGTAAACAC	Binds within *luxC* coding region, used for sequencing insertion site of mTn5*luxkan2* transposon
BA1090	GAATGTATGTCCTGCGTCTTGAGTA	Binds within *luxC* coding region; used for sequencing insertion site of mTn5*luxkan2* transposon
BA2276	CAGTAAGTATGAGGGATATAGACTTTTTCACCTG	Binds upstream of *E. cloacae sdiA* gene
BA2277	GAGCACACCTGAATTTGCCACTGCCGAGAATAAC	Binds downstream of *E. cloacae sdiA* gene
BA2219	CTGTCTCTTATACACATCTGTGTAGGCTGGAGCTGCTTC	Binds the P1 region of pCLF4.pCLF3, pKD3, pKD4 for amplification of the FRT-cam/kan-FRT cassette with ME sequences
BA2220	CTGTCTCTTATACACATCTCATATGAATATCCTCCTTAG	Binds the P2 region of pCLF4.pCLF3, pKD3, pKD4 for amplification of the FRT-cam/kan-FRT cassette with ME sequences
BA1598	GATCTTCCGTCACAGGTAGG	Binds within the chloramphenicol resistance marker (C2)
BA2343	GCGTTCAATTTGCTCCAGATGCCGCTTCTGG	Binds upstream of *E. cloace sdiA* gene
IPCRF	TTTTGGTGATAATAGTGTTTACCTGCC	Forward primer for inverse PCR with miniTn5
IPCRR	TTTTTTTAGTCATACGTATCCTCCAAGCC	Reverse primer for inverse PCR with miniTn5
BA2447	GAAAAGGATAGCACAGGATCTGAGAAAGG	Primer binds within ENC_14960; used with BA1090 for identification of insertion site
BA2448	GCCACAGCGTGAATTGCAGGTGCTGGATGCGC	Primer binds within ENC_40870; used with BA1090 for identification of insertion site

Not all insertion points were identified using Sanger genomic DNA sequencing. The rest were identified using inverse PCR. Genomic DNA was digested with *Nla*III (NEB) for 3 h at 37°C. The enzyme was inactivated for 20 min at 65°C, then T4 DNA ligase (NEB) was added to the digestion reaction in a total reaction volume of 200 μL at 16°C overnight. The ligation reaction was purified using the QiaQuick PCR purification kit (Qiagen), digested with *Xmn*I for 3 h at 37°C, then heat inactivated at 65°C for 20 min. This digest was used in a PCR reaction using the primers IPCRF and IPCRR (Table 2) using *Taq* DNA polymerase (NEB). The PCR product was sequenced using the IPCRF primer at the Plant Microbe Genomics Facility at The Ohio State University.

### Liquid and motility agar assays for *lux* fusions

Strains were grown in LB kan for *sdiA*+ strains or LB kan cam for *sdiA* mutants, and grown at 37°C shaking overnight. They were then subcultured 1:100 in triplicate into either LB broth or LB motility agar containing the appropriate antibiotics and either 1 μM oxoC6 or 0.1% EA as the solvent control and then placed in the well of a black clear bottom 96-well plate. The plate was grown with shaking at 37°C and time points were taken by placing the 96-well plate in the Wallac Victor plate reader. For broth cultures, both OD_590_ and luminescence were measured. For the motility agar assays, only the luminescence was measured. For the AHL concentration sensitivity assay, an *sdiA*-regulated fusion was assayed in LB broth or motility agar with 10-fold dilutions of either oxoC6 or oxoC8 starting at a concentration of 1 μM. AHL-dependent changes in gene expression were analyzed using a Two-Way ANOVA over the time course. *P*-values were marked accordingly (≤0.05 = ^*^, ≤0.005 = ^**^, ≤0.0005 = ^***^, ≤0.00005 = ^****^). The *sdiA*-dependent changes in gene expression were larger than the AHL-dependent changes and were statistically significant (*p* ≤ 0.00005) for all fusions in all growth conditions for at least one time point.

### Construction of an *sdiA* mutant of *E. cloacae*, using a new suicide vector and transposon mutagenesis strategy

**Figure 1:**
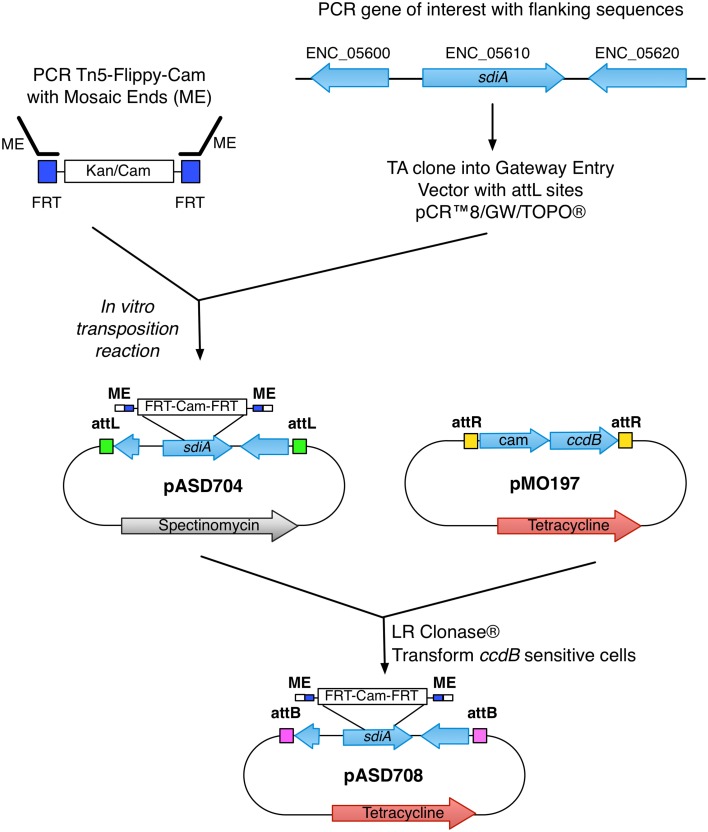
**Generation of the ***sdiA*** mutant in ***E. cloacae*****. Schematic of the construction and generation of the *sdiA* mutant in *E. cloacae* using the mTn5-FC and novel suicide vector pMO197.

The new suicide vectors are derivatives of pDMS197 (Edwards et al., 1998) and pGP704 (Miller and Mekalanos, 1988), respectively, that have been modified for the Gateway cloning system of Invitrogen, which uses phage attachment sites for *in vitro* recombination reactions. To modify these vectors, a blunt-ended DNA fragment (Reading Frame Cassette C) obtained from Invitrogen containing *attR*-cam^r^-*ccdB*-*attR* was ligated into the *Sma*I site of pGP704 and pDMS197, resulting in pMO704 and pMO197, respectively (Figure 1).

With the suicide vectors completed, the *sdiA* gene of *E. cloacae* was amplified by PCR using primers BA2276 and BA2277 (Table 2) and cloned into the Gateway entry vector pCR8/GW/TOPO creating pASD704. This vector has *attL* sites flanking the inserted PCR product. Mutations were created in this cloned *E. cloacae sdiA* gene using *in vitro* transposon mutagenesis. We utilized a new mTn*5* derivative that we named mTn5-FC where FC stands for flippy-cam (Figure 1). Essentially, the DNA sequences of the optimized mosaic ends of Tn*5* (Goryshin and Reznikoff, 1998) are appended to PCR primers (BA2219 and BA2220) that are used to amplify the FRT-cam^r^-FRT cassette from pCLF3 (Santiviago et al., 2009). The resulting PCR product is the transposon. To mutagenize the *sdiA* gene on pASD704, plasmid DNA and transposon DNA were mixed in the presence of transposase enzyme in an *in vitro* transposition reaction. This reaction mix was then transformed into DH5αλ pir and plated on LB spec cam. The resulting colonies were screened for transposon insertions in the *sdiA* gene using PCR with primers BA2276 and BA1598. One insertion of interest, named *sdiA32*::mTn5-FC (pASD706), was then used in an “LR” cloning reaction to recombine the mutated *sdiA* gene into the new pMO197 suicide vector by transforming BW20767, creating pASD708. This vector was then mobilized into each of the *E. cloacae* strains, selecting for replacement of the wild-type *sdiA* allele with the mutant allele, which was verified by PCR using primers BA2343 and BA1598.

## Results

### Identification of AHL-responsive transcriptional fusions in *E. cloacae*

A murine isolate of *Enterobacter cloacae* strain, JLD400, was found to be genetically tractable and sensitive to all antibiotics tested except ampicillin (Ali et al., 2014). It is easily electroporated and readily serves as a recipient in RP4-mediated conjugation. A mutant resistant to nalidixic acid was isolated after passage on LB with nalidixic acid (JLD401, Table 1).

In order to identify genes that are regulated in response to AHLs in *E. cloacae*, we constructed random transcriptional fusions to the luciferase genes of *Photorhabdus luminescens* (*luxCDABE)* using the transposon mTn*5*-*luxCDABE* (Winson et al., 1998). Previous screens for *sdiA*-regulated targets in *Salmonella* have utilized plasmid-borne *sdiA*, which bypasses the AHL requirement, but this approach has been shown to have pleotropic effects in *E. coli* (Ahmer, 2004; Dyszel et al., 2010b). Therefore, we took an alternate strategy in which we mutagenized the wild-type strain with the mTn5-*luxCDABE* transposon while *sdiA* remained in its native position in the chromosome and screened 10,000 mutants for responsiveness to synthetic AHL (oxoC6, which is detected by SdiA of *E. coli* and *Salmonella*). Seventeen insertions were identified that demonstrated an increase in luminescence greater than 2.5-fold.

**Figure 2:**
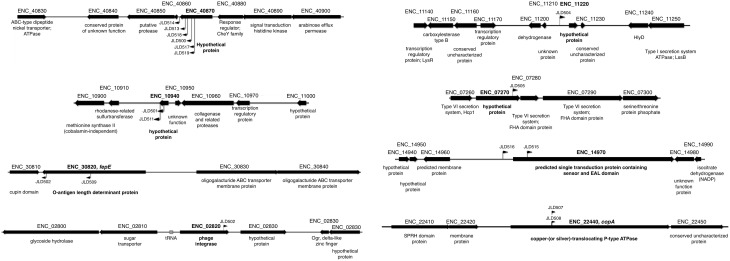
**Insertion points of AHL-regulated genes in ***E. cloacae*****. Diagram of the location of AHL-regulated mTn5-*luxCDABE* insertions. The mTn5-*luxCDABE* insertion points and orientation are indicated by flags.

The transposon insertion point was identified for all 17 insertions using either of two methods: (1) sequencing genomic DNA using two different sequencing primers that bind within the transposon sequence and are oriented outward, or (2) inverse PCR and subsequent sequencing of the product. After identification of the transposon insertion sites, a confirmatory PCR was performed using a primer within the transposon and another within the putative AHL-responsive gene. A positive PCR reaction confirms that the transposon insertion is in the correct location, but we also sequenced the resulting PCR product to further define the transposon insertion site. All 17 AHL-responsive fusions were located within 8 unique genes (Table 3, Figure 2). The genome sequence of our *Enterobacter cloacae* isolate is not known, but BLAST searches revealed that the majority of these sequences were most similar to the genome sequence of *Enterobacter cloacae* subspecies *cloacae* NCTC 9394 (FP929040). The transposon insertions of six strains (JLD500, JLD513, JLD514, JLD517, JLD518, and JLD519) were within ENC_40870, which encodes a hypothetical protein only present in *Enterobacter cloacae*. JLD501 and JLD511 each contained an insertion in ENC_10940, which encodes a hypothetical protein with a secretion signal predicted by SignalP within the first 20 amino acids (Petersen et al., 2011). JLD502 and JLD509 each contained an insertion in ENC_30820, which encodes a homolog of FepE, a protein that increases the length of O antigen chains (Murray et al., 2003; Crawford et al., 2012, 2013). JLD504 has an insertion within the intergenic region of ENC_11220, which encodes a hypothetical protein. JLD505 contained an insertion in ENC_07270, which encodes a hypothetical protein within an operon that encodes a putative type VI secretion system (Durand et al., 2014; Li et al., 2015). JLD506 contains an insertion in ENC_02820, which encodes a prophage integrase. JLD507 and JLD508 each contained an insertion in ENC_22440, which encodes a homolog of CopA, a putative copper-translocating P-type ATPase (Rensing and Grass, 2003; Osman and Cavet, 2011). JLD515 and JLD516 have insertions within the promoter region of ENC_14970, encoding a putative signal transduction protein containing a sensor and diguanylate phosphodiesterase (EAL) domain (Römling et al., 2013).

**Table 3:** **AHL-responsive, *sdiA*-dependent fusions identified in *E. cloacae***.

			***sdiA*-dependent, AHL-dependent fold change in reporter expression^a^,^b^**					
**Gene Hits**	**Insertion Site**	**Fusions**	**37°C shaking**	**30°C shaking**	**37°C standing**	**30°C standing**	**37.C motility agar**	**37.C motility agar**
ENC_40870	Hypothetical protein	JLD500						
		JLD513	67.7	**317.4**	32.2	49.9	128.0	236.2
		JLD514						
		JLD517	5.7	2.7	9.3	3.4	**9.9**	2.2
		JLD518						
		JLD519						
ENC_10940	Hypothetical protein	JLD501	45.0	28.6	48.8	13.4	41.9	**50.9**
		JLD511	**27.4**	9.2	19.3	8.6	9.1	8.1
ENC_30820	*fepE*	JLD502	46.9	**58.2**	41.6	25.9	40.6	50.7
		JLD509	**24.0**	7.4	13.0	8.2	5.7	4.3
ENC_11220	Intergenic region	JLD504	72.4	82.5	223.8	45.7	**15975.8**	6912.2
			22.4	33.6	41.9	18.2	**178.6**	37.3
ENC_07270	Hypothetical protein in a putative type VI	JLD505	44.6	82.3	90.4	79.5	**233.1**	155.3
	secretion system operon		1.9	2.0	**3.4**	1.3	2.5	2.0
ENC_02820	Phage integrase	JLD506	13.1	8.6	**14.7**	12.0	5.7	6.6
ENC_22440	*copA*	JLD507 JLD508	8.6	5.1	**9.3**	5.9	2.1	8.6

a *Top number is the largest fold change in sdiA-dependent expression throughout the time course for each fusion. The bottom number is the largest fold change in AHL-dependent expression throughout the time course for each fusion. The highest sdiA-dependent or AHL-dependent fold change is indicated for each fusion in bold*.

b *For fusions ENC_02820 and ENC_22440 only the sdiA-dependent fold change is displayed (calculated from the cultures that included AHL). Neither fusion exhibited statistically significant AHL-dependent changes in expression*.

**Figure 3:**
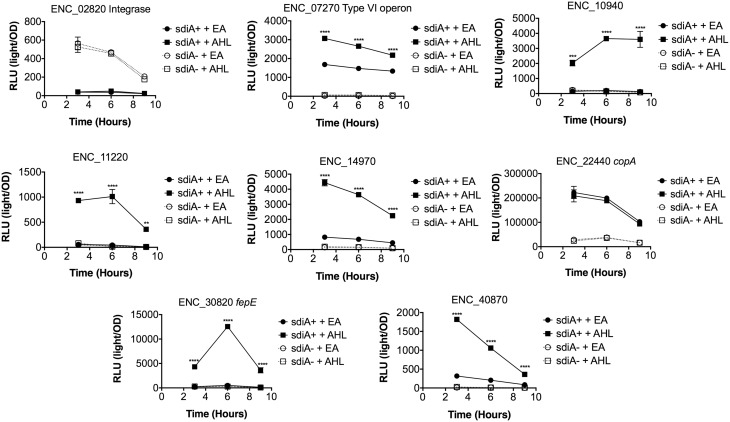
**Regulation of AHL-regulated genes in ***E. cloacae*** in LB broth at 37°C shaking**. Expression of mTn5*luxCDABE* fusion strains in either the wild-type (closed symbols) or *sdiA* mutant backgrounds (open symbols) with either 1 μM oxoC6 (squares) or 0.1% ethyl acetate (EA) solvent control (circles) in LB broth. Luminescence is reported in relative light units (light/OD_590_). Data was collected at 3, 6, and 9 h time points. All data points are the average of three technical replicates and error bars indicate SEM. This is a representative graph of three independent biological replicates. The statistical significance of AHL-dependent changes in gene expression are designated with *p*-values (≤0.05 = ^*^, ≤0.005 = ^**^, ≤0.0005 = ^***^, ≤0.00005 = ^****^). The *sdiA*-dependent changes in gene expression were larger than the AHL-dependent changes and were statistically significant (*p* = 0.00005) for all fusions in all growth conditions for at least one time point. The *sdiA*^+^ and *sdiA* mutant strains for each fusion are indicated in parentheses: ENC_02820 (JLD506/ASD506), ENC_07270 (JLD505/ASD505), ENC_10940 (JLD501/ASD501), ENC_11220 (JLD504/ASD504), ENC_14970 (JLD515/ASD515), ENC_22440 (JLD508/ASD508), ENC_30820 (JLD502/ASD502), and ENC_40870 (JLD500/ASD500).

**Figure 4:**
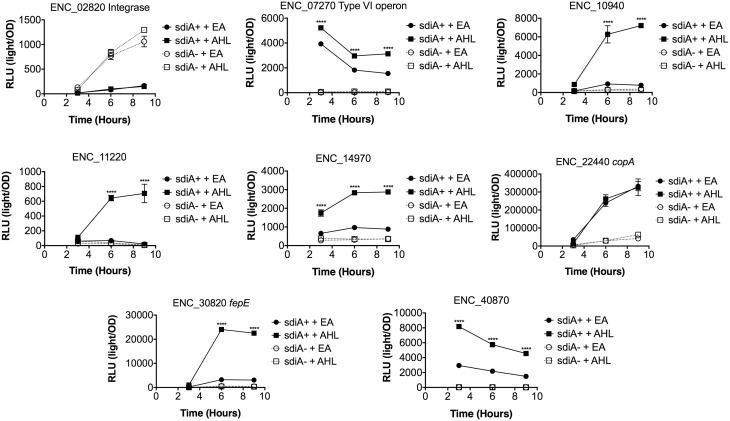
**Regulation of AHL-regulated genes in ***E. cloacae*** in LB broth at 30°C shaking**. Expression of mTn5*luxCDABE* fusion strains in either the wild-type (closed symbols) or *sdiA* mutant backgrounds (open symbols) with either 1 μM oxoC6 (squares) or 0.1% ethyl acetate (EA) solvent control (circles) in LB broth. Luminescence is reported in relative light units (light/OD_590_). Data was collected at 3, 6, and 9 h time points. All data points are the average of three technical replicates and error bars indicate SEM. This is a representative graph of three independent biological replicates. The statistical significance of AHL-dependent changes in gene expression are designated with *p*-values (≤0.05 = ^*^, ≤0.005 = ^**^, ≤0.0005 = ^***^, ≤0.00005 = ^****^). The *sdiA*-dependent changes in gene expression were larger than the AHL-dependent changes and were statistically significant (*p* = 0.00005) for all fusions in all growth conditions for at least one time point. The *sdiA*^+^ and *sdiA* mutant strains for each fusion are indicated in parentheses: ENC_02820 (JLD506/ASD506), ENC_07270 (JLD505/ASD505), ENC_10940 (JLD501/ASD501), ENC_11220 (JLD504/ASD504), ENC_14970 (JLD515/ASD515), ENC_22440 (JLD508/ASD508), ENC_30820 (JLD502/ASD502), and ENC_40870 (JLD500/ASD500).

**Figure 5:**
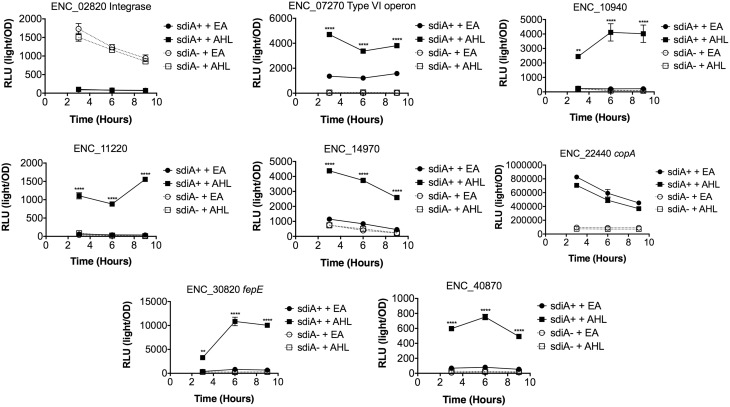
**Regulation of AHL-regulated genes in ***E. cloacae*** in LB broth at 37°C standing**. Expression of mTn5*luxCDABE* fusion strains in either the wild-type (closed symbols) or *sdiA* mutant backgrounds (open symbols) with either 1 μM oxoC6 (squares) or 0.1% ethyl acetate (EA) solvent control (circles) in LB broth. Luminescence is reported in relative light units (light/OD_590_). Data was collected at 3, 6, and 9 h time points. All data points are the average of three technical replicates and error bars indicate SEM. This is a representative graph of three independent biological replicates. The statistical significance of AHL-dependent changes in gene expression are designated with *p*-values (≤0.05 = ^*^, ≤0.005 = ^**^, ≤0.0005 = ^***^, ≤0.00005 = ^****^). The *sdiA*-dependent changes in gene expression were larger than the AHL-dependent changes and were statistically significant (*p* ≤ 0.00005) for all fusions in all growth conditions for at least one time point. The *sdiA*^+^ and *sdiA* mutant strains for each fusion are indicated in parentheses: ENC_02820 (JLD506/ASD506), ENC_07270 (JLD505/ASD505), ENC_10940 (JLD501/ASD501), ENC_11220 (JLD504/ASD504), ENC_14970 (JLD515/ASD515), ENC_22440 (JLD508/ASD508), ENC_30820 (JLD502/ASD502), and ENC_40870 (JLD500/ASD500).

**Figure 6:**
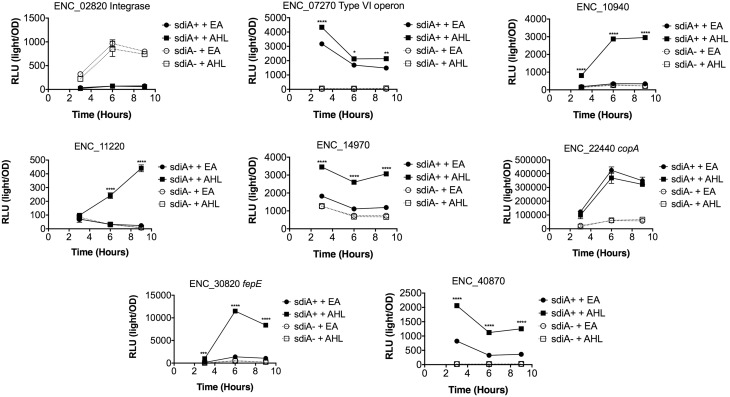
**Regulation of AHL-regulated genes in ***E. cloacae*** in LB broth at 30°C standing**. Expression of mTn5*luxCDABE* fusion strains in either the wild-type (closed symbols) or *sdiA* mutant backgrounds (open symbols) with either 1 μM oxoC6 (squares) or 0.1% ethyl acetate (EA) solvent control (circles) in LB broth. Luminescence is reported in relative light units (light/OD_590_). Data was collected at 3, 6, and 9 h time points. All data points are the average of three technical replicates and error bars indicate SEM. This is a representative graph of three independent biological replicates. The statistical significance of AHL-dependent changes in gene expression are designated with *p*-values (≤0.05 = ^*^, ≤0.005 = ^**^, ≤0.0005 = ^***^, ≤0.00005 = ^****^). The *sdiA*-dependent changes in gene expression were larger than the AHL-dependent changes and were statistically significant (*p* = 0.00005) for all fusions in all growth conditions for at least one time point. The *sdiA*^+^ and *sdiA* mutant strains for each fusion are indicated in parentheses: ENC_02820 (JLD506/ASD506), ENC_07270 (JLD505/ASD505), ENC_10940 (JLD501/ASD501), ENC_11220 (JLD504/ASD504), ENC_14970 (JLD515/ASD515), ENC_22440 (JLD508/ASD508), ENC_30820 (JLD502/ASD502), and ENC_40870 (JLD500/ASD500).

**Figure 7:**
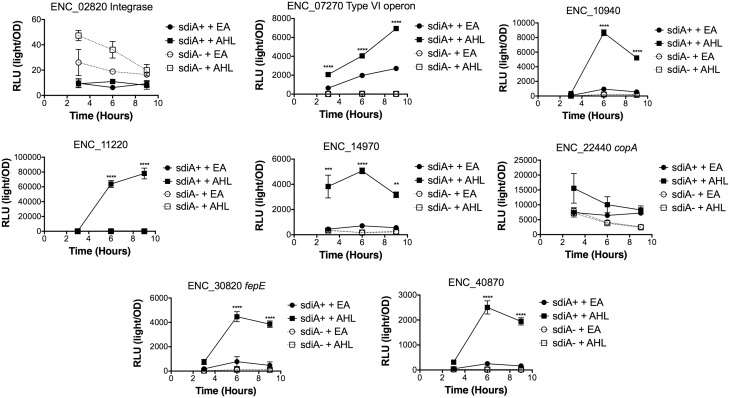
**Regulation of AHL-regulated genes in *E. cloacae* in motility agar at 37°C**. Expression of mTn5*luxCDABE* fusion strains in either the wild-type (closed symbols) or *sdiA* mutant backgrounds (open symbols) with either 1 μM oxoC6 (squares) or 0.1% ethyl acetate (EA) solvent control (circles) in LB broth. Luminescence is reported in relative light units (light/OD_590_). Data was collected at 3, 6, and 9 h time points. All data points are the average of three technical replicates and error bars indicate SEM. This is a representative graph of three independent biological replicates. The statistical significance of AHL-dependent changes in gene expression are designated with *p*-values (≤0.05 = ^*^, ≤0.005 = ^**^, ≤0.0005 = ^***^, ≤0.00005 ≤ ^****^). The *sdiA*-dependent changes in gene expression were larger than the AHL-dependent changes and were statistically significant (*p* = 0.00005) for all fusions in all growth conditions for at least one time point. The *sdiA*^+^ and *sdiA* mutant strains for each fusion are indicated in parentheses: ENC_02820 (JLD506/ASD506), ENC_07270 (JLD505/ASD505), ENC_10940 (JLD501/ASD501), ENC_11220 (JLD504/ASD504), ENC_14970 (JLD515/ASD515), ENC_22440 (JLD508/ASD508), ENC_30820 (JLD502/ASD502), and ENC_40870 (JLD500/ASD500).

**Figure 8:**
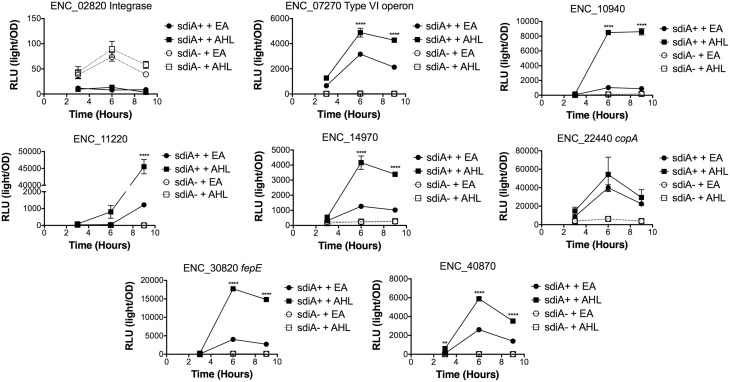
**Regulation of AHL-regulated genes in ***E. cloacae*** in motility agar at 30°C**. Expression of mTn5*luxCDABE* fusion strains in either the wild-type (closed symbols) or *sdiA* mutant backgrounds (open symbols) with either 1 μM oxoC6 (squares) or 0.1% ethyl acetate (EA) solvent control (circles) in LB broth. Luminescence is reported in relative light units (light/OD_590_). Data was collected at 3, 6, and 9 h time points. All data points are the average of three technical replicates and error bars indicate SEM. This is a representative graph of three independent biological replicates. The statistical significance of AHL-dependent changes in gene expression are designated with *p*-values (≤0.05 = ^*^, ≤0.005 = ^**^, ≤0.0005 = ^***^, ≤0.00005 = ^****^). The *sdiA*-dependent changes in gene expression were larger than the AHL-dependent changes and were statistically significant (*p* ≤ 0.00005) for all fusions in all growth conditions for at least one time point. The *sdiA*^+^ and *sdiA* mutant strains for each fusion are indicated in parentheses: ENC_02820 (JLD506/ASD506), ENC_07270 (JLD505/ASD505), ENC_10940 (JLD501/ASD501), ENC_11220 (JLD504/ASD504), ENC_14970 (JLD515/ASD515), ENC_22440 (JLD508/ASD508), ENC_30820 (JLD502/ASD502), and ENC_40870 (JLD500/ASD500).

**Figure 9:**
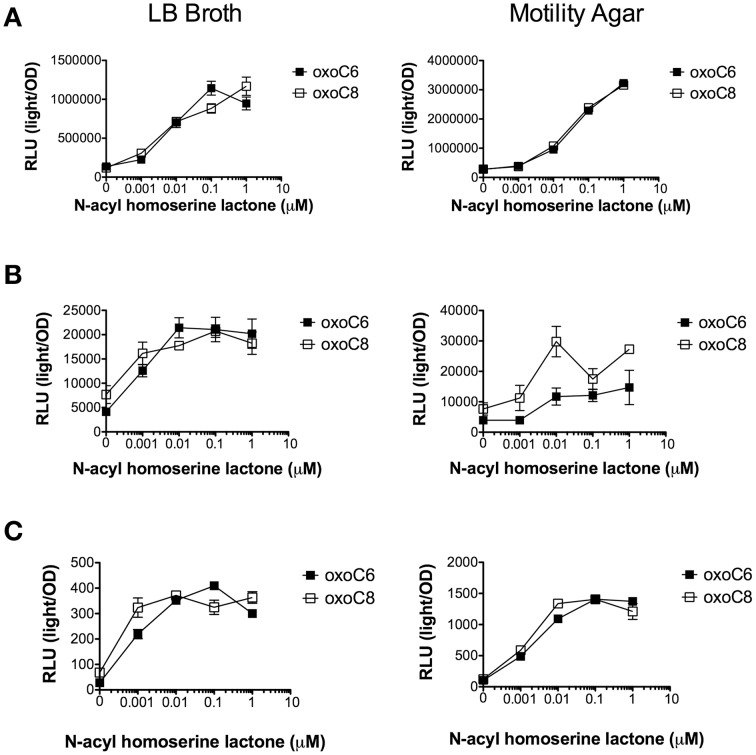
**AHL concentration dependent response of ***Salmonella***, ***E. coli***, and ***Enterobacter***. (A)** Expression of *srgE-luxCDABE* fusion (pJNS25) in *S*. Typhimurium 14028. **(B)** Expression of the *gadW*::Tn5*luxCDABE* fusion in *E. coli* K-12 AL4001. **(C)** Expression of the ENC_40870::Tn5*luxCDABE* fusion in *E. cloacae* JLD500. Assays were done in either LB liquid broth with shaking or motility agar (0.3% agar) at 37°C. Relative light units (light/OD_590_) after 6 h of growth are indicated for oxoC6 (black squares) or oxoC8 (open squares). All data points are the mean of three biological replicates and error bars indicate SEM.

In *Salmonella*, we have observed differences in the behavior of *sdiA*-regulated fusions at 30°C compared to 37°C, and in motility agar compared to broth or agar plates (Smith and Ahmer, 2003). More ligand-independent SdiA activity is observed at lower temperatures, and more activity in general is observed in motility agar than in broth or agar plates. For *E. cloacae*, therefore, we chose one representative fusion-containing strain for each AHL-responsive locus identified, and tested these representatives under each of these conditions (Figures 3–8). Unlike *sdiA*-regulated fusions in *Salmonella*, the *E. cloacae* fusions did not become more ligand-independent at 30°C compared to 37°C. Instead, some fusions were largely ligand-independent under all conditions, while the remainder were ligand-dependent under all conditions. We also tested the response of these fusions to a series of AHL concentrations using oxoC6 and oxoC8. The AHL detection limits of *E. cloacae* are similar to those of *E. coli* and *Salmonella* (Figure 9).

### AHL-responses are *sdiA*-dependent

We hypothesized that the AHL-dependent responses of the *lux* fusions were dependent on the *sdiA* gene encoded on the chromosome of *E. cloacae*. To test this hypothesis, we constructed an *sdiA* mutation in each of the mTn*5-luxCDABE* fusion strains using two new suicide vectors and a new transposon mutagenesis strategy (see Materials and Methods). Indeed, mutation of *sdiA* in each fusion strain eliminated any responsiveness to AHL in both liquid culture and motility agar (Table 3). In one case, *sdiA* in *E. cloacae* acts as a negative regulator of expression. We observed that *sdiA* is required for repression of ENC_02820 since the *sdiA* mutant strain produced more light than the wild-type strain regardless of the presence of AHL, in all of the conditions tested (Figures 3–8).

## Discussion

SdiA is a LuxR homolog that detects the AHLs produced by other bacteria (Michael et al., 2001; Smith and Ahmer, 2003). To date, SdiA regulon members in *S. enterica* serovar Typhimurium*, E. coli* K-12, EHEC and a plant-pathogenic isolate of *Enterobacter cloacae* have been described (Ahmer et al., 1998; Kanamaru et al., 2000; Wei et al., 2001; Suzuki et al., 2002; Smith and Ahmer, 2003; Van Houdt et al., 2006; Lee et al., 2007; Ghosh et al., 2009; Dyszel et al., 2010b; Hughes et al., 2010; Sharma et al., 2010; Shankar et al., 2012; Sharma and Bearson, 2013). Here we report the identification of AHL-responsive and *sdiA*-dependent genes in a mouse isolate of *E. cloacae*. We have previously shown that this *E. cloacae* isolate is not pathogenic and competes with *Salmonella* for colonization of mice (Ali et al., 2014). To identify AHL-responsive genes in this organism, we used a transposon-based genetic screen in which the expression of luciferase by individual mTn*5*-*luxCBADE* mutants was measured in the presence and absence of AHL. The *sdiA* gene was then mutated in each strain, and the response of every fusion was found to be *sdiA*-dependent (Figures 3–8). This suggests that SdiA is the only AHL receptor in this isolate of *E. cloacae*.

In *Salmonella*, there is very little SdiA activity in the absence of AHL at 37°C, although some is observed at 30°C (Smith and Ahmer, 2003; Sabag-Daigle et al., 2012). In *E. coli* there seems to be more SdiA activity in the absence of AHL (Dyszel et al., 2010b; Hughes et al., 2010; Sperandio, 2010b). Other work in *E. coli* has shown that SdiA binds target genes *in vivo* in the absence of AHL (Ishihama et al., 2014; Shimada et al., 2014). AHL-independent activity of SdiA was also noted with some, but not all, plasmids used as AHL biosensors in *E. coli* (Lindsay and Ahmer, 2005). However, the *E. cloacae* regulon identified here is very unusual in that SdiA is demonstrating high levels of AHL-independent activity for some fusions but not others. For instance, substantial AHL-independent SdiA activity is observed with ENC_22440 and ENC_07270 (Figures 3–8). Another fusion was repressed by *sdiA* and this was also independent of AHL (ENC_02820) (Figures 3–8). It appears that we were fortunate to identify these particular fusions using AHL-responsiveness as the first screen. The LuxR homolog TraR requires AHL for proper structural folding in order to oligomerize into a fully functional dimer structure capable of binding its target promoters (Zhu and Winans, 2001). However, the ligand-independent activity of SdiA suggests that SdiA is properly folded and able to bind target promoters even in the absence of AHL. This may be due to the folding of SdiA around endogenous 1-octanoyl-*rac*-glycerol (Nguyen et al., 2015). The mechanistic differences between ligand-dependent and -independent regulation of genes by SdiA is an interesting topic for further studies.

**Figure 10:**
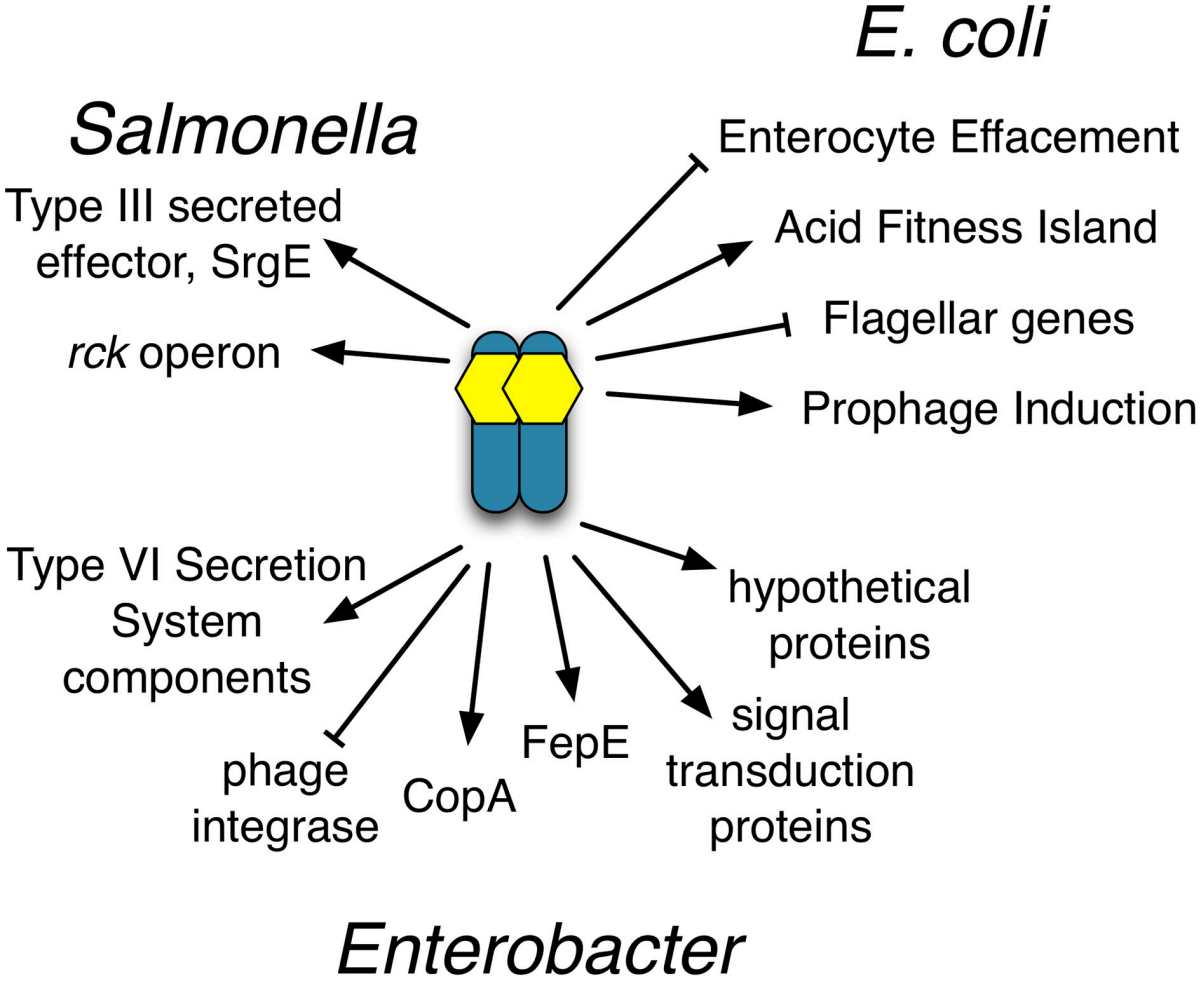
**Compilation of the SdiA regulon members in ***Salmonella***, EHEC and ***Enterobacter*****. Diagram of known SdiA regulon members in *Salmonella*, EHEC and *Enterobacter*. Arrows indicate whether SdiA increases the expression of the regulon member and blunt arrows indicate SdiA decreases the expression of the regulon member, either directly or indirectly.

The role(s) for the SdiA regulon in this isolate of *E. cloacae* is unclear (Figures 2, 10). The *fepE, copA*, and type VI secretion genes could be envisioned to have direct interactions with the host, direct interactions with other microbes, or in general survival in the intestinal tract. Alternatively, these genes may play a role outside the host in other environments. In *E. coli*, the induction of lambda prophage is enhanced by AHL in an *sdiA*-dependent manner (Ghosh et al., 2009). The fusion that was repressed by *sdiA* in this study encodes a putative phage integrase, although it does not appear to be encoded within a prophage (Figure 2). It would be interesting to determine if SdiA plays a role in phage biology of *E. cloacae*.

In an isolate of *E. cloacae* that promotes the growth of rice roots, SdiA represses biofilm formation and rice root colonization (Shankar et al., 2012). This phenotype is at least partially due to the *sdiA*-dependent repression of the genes encoding curli fimbriae (Shankar et al., 2012). Repression of biofilm formation has been observed in *E. coli* as well (Lee et al., 2009; Sharma et al., 2010). Interestingly, we did not isolate curli genes in this study. It is not known if curli genes are not regulated by *sdiA* in this isolate, or if we simply missed them, either by chance or due to growth conditions. It would be interesting to determine if the genes identified in this study are regulated by *sdiA* in the *E. cloacae* plant isolate and to determine if the *sdiA* regulon has diverged between the plant and mouse isolates, or if the regulon has remained largely the same.

### Conflict of interest statement

The authors declare that the research was conducted in the absence of any commercial or financial relationships that could be construed as a potential conflict of interest.
